# An Efficient Deep Learning Model to Detect COVID-19 Using Chest X-ray Images

**DOI:** 10.3390/ijerph19042013

**Published:** 2022-02-11

**Authors:** Somenath Chakraborty, Beddhu Murali, Amal K. Mitra

**Affiliations:** 1 School of Computing Sciences and Computer Engineering, The University of Southern Mississippi, Hattiesburg, MS 39406, USA; somenath.chakraborty@usm.edu (S.C.); beddhu.murali@usm.edu (B.M.); 2 Department of Epidemiology and Biostatistics, School of Public Health, College of Health Sciences, Jackson State University, Jackson, MS 39213, USA

**Keywords:** COVID-19, SARS-CoV-2, chest X-ray, Deep Learning Model

## Abstract

The tragic pandemic of COVID-19, due to the Severe Acute Respiratory Syndrome coronavirus-2 or SARS-CoV-2, has shaken the entire world, and has significantly disrupted healthcare systems in many countries. Because of the existing challenges and controversies to testing for COVID-19, improved and cost-effective methods are needed to detect the disease. For this purpose, machine learning (ML) has emerged as a strong forecasting method for detecting COVID-19 from chest X-ray images. In this paper, we used a Deep Learning Method (DLM) to detect COVID-19 using chest X-ray (CXR) images. Radiographic images are readily available and can be used effectively for COVID-19 detection compared to other expensive and time-consuming pathological tests. We used a dataset of 10,040 samples, of which 2143 had COVID-19, 3674 had pneumonia (but not COVID-19), and 4223 were normal (not COVID-19 or pneumonia). Our model had a detection accuracy of 96.43% and a sensitivity of 93.68%. The area under the ROC curve was 99% for COVID-19, 97% for pneumonia (but not COVID-19 positive), and 98% for normal cases. In conclusion, ML approaches may be used for rapid analysis of CXR images and thus enable radiologists to filter potential candidates in a time-effective manner to detect COVID-19.

## 1. Introduction

The Severe Acute Respiratory Syndrome Coronavirus-2 (SARS-CoV-2) has created the greatest pandemic of COVID-19 in nearly a century and has already traumatized the whole world [1,2,3]. The pandemic reportedly started in the City of Wuhan, China in late December, 2019 [1,2,3]. In two years, as of 9 December 2021, over 268 million cases and 5.3 million deaths have been caused by the pandemic worldwide [4]. In the United States alone, there were over 50.4 million cases and 814,000 deaths caused by COVID-19. Lung involvement is the most common noticeable manifestation of the disease, ranging from asymptomatic disease or mild pneumonia, to severe disease associated with hypoxia, critical disease associated with shock, respiratory failure and multi-organ failure or death [5]. An early and accurate detection of the COVID-19 virus is one of the cornerstones for containing the spread and minimizing the death toll of the disease. 

In an early stage of the outbreak, due to the lack of knowledge of the virus genome, the detection of the disease was rudimentary. COVID-19 RT-PCR is a real-time reverse transcription polymerase chain reaction test. It offers a qualitative detection of nucleic acid from SARS-CoV-2 which is present in the respiratory specimens during the infection [6]. RT-PCR is used as the gold standard for the detection of COVID-19. Many challenges, however, still remain in the COVID-19 detection research. The RT-PCR sensitivity rate is 60–70%, and the test is expensive [7]. Antigen detection tests, or commonly known as rapid COVID tests, are fairly accurate in detecting positive cases but the false negative rate of the test is high and the sensitivity of the test is low (60.5%) [7]. 

Two other simpler methods in the detection of COVID-19 include the use of chest X-ray (CXR) and chest CT-scan [8,9,10,11,12,13,14,15,16]. Artificial Intelligence (AI) can be used in medical image processing where we can leverage AI for automatic detection and further guidance. We propose to detect COVID-19 from CXR images using an AI approach called a Deep Learning Model (DLM). Deep Learning methods have revolutionized the field of AI, and due to their potential application in medical diagnosis and prognosis systems, millions of human lives could be saved [17]. Radiographic images such as CXR are inexpensive and less time consuming to produce as results compared to other clinical or laboratory modalities. COVID-19 positive is characterized primarily by patches of ground-glass opacity and consolidations [14]. Therefore, CXR dataset is useful to test algorithms for detecting COVID-19 and other pulmonary disorders. Successful machine learning approaches could allow for rapid evaluations of chest X-ray images and thus enable radiologists to filter potential candidates in a time-effective manner [15,16]. Studies using deep neural networks have shown the effectiveness of the method in the diagnosis of pneumonia [15]. Another study of deep artificial neural networks which used more than 470,300 chest X-rays from hospitalized patients in three hospitals was able to detect abnormal cases with a positive predictive value of 94% [16]. In the later study, the average reporting delay was reduced by 76% from 11.2 to 2.7 days for critical X-ray findings (*p* < 0.001) in the model compared with historical data [16].

According to reports from the Centers for Disease Control and Prevention (CDC) [18], the Delta variant of the coronavirus (B.1.617.2) is spreading more rapidly and causing more infections compared with the original SARS-CoV-2 strain. Currently, the use vaccines remains the most effective method to reduce the risk of severe illnesses, transmission, hospitalization, and deaths due to COVID-19. However, CDC cautioned that effectiveness of the vaccines against new variants that may arise, including the new coronavirus variant Omicron (B.1.1.529) is not clear at this point in time [19]. 

Detection of COVID-19 using CXR has certain advantages over other methods. Currently, two types of diagnostic tests—molecular (RT-PCR) tests for the detection of the virus’s genetic material, and antigen tests for the detection of a specific viral agent—are available at the point of care [20]. For many countries, these laboratory tests continue to be costly, time consuming, and are not readily available for a mass population use. CXR image analysis shows promise in making the process of diagnosis faster, cheaper and more readily accessible compared to the other procedures [8,9,14,21]. Because of the multiple mutations of the virus and the emergence of newer variants such as the Delta variant and the Omicron coronavirus variant (B.1.1.529), the search for an early and accurate method of detection of COVID-19 is crucial. If an automated AI model using chest X-rays is readily available to the health professionals, it will be a substantial improvement in the clinical management of the disease. 

For these purposes, we aimed to evaluate the effectiveness of the Deep Learning Model (DLM) using CXR in screening COVID-19 cases. We compared our results with previously reported models in predicting COVID-19.

## 2. Materials and Methods

### 2.1. Data Acquisition

**Figure 1:**
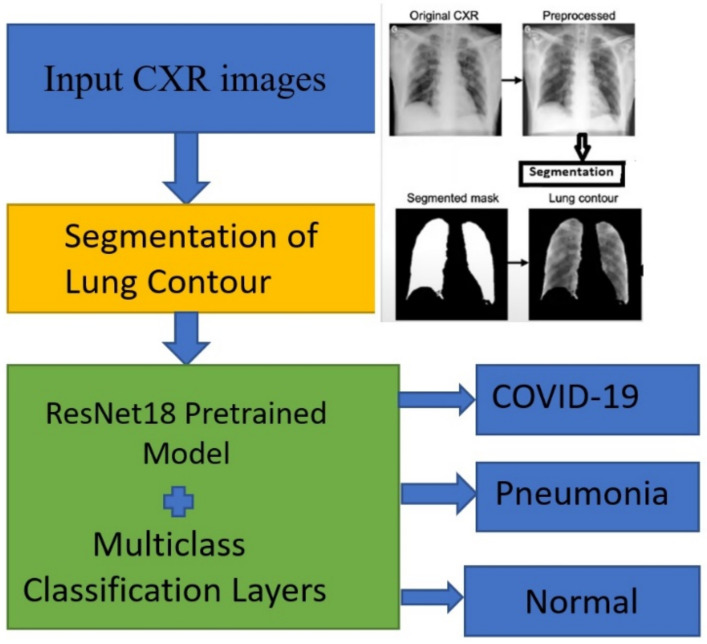
A Block Diagram Representation of the Deep Learning Model (DLM).

The research was conducted from September 2020 to December 2021. To design a machine learning model as depicted in Figure 1, it takes a few basic steps to follow, of which the most important and formidable task was to gather a sufficient number of reliable sample data. The data collection was done from open repositories on the Internet including Kaggle and GitHub where CXR data are contributed from different hospitals and other institutions around the world [22,23,24,25]. The data consisted of: (1) CXR images of COVID-19 infected patients, (2) CXR images of samples with pneumonia but not having COVID-19 infections, and (3) normal CXR images. Our model used medical image segmentation to get more accurate feature vector component which was then processed by deep neural network.

### 2.2. Preprocessing and Image Segmentation

After data acquisition, we used the following preprocessing steps with the CXR images. Firstly, we discarded CXR images that did not have labels from radiologists. Then after splitting into training, validation and test datasets, we applied multiple transformations including annotation, labeling, normalization and augmentation to each image in each dataset [26]. Preprocessing makes the data more robust and better suitable for Deep Learning Models. 

**Figure 2:**
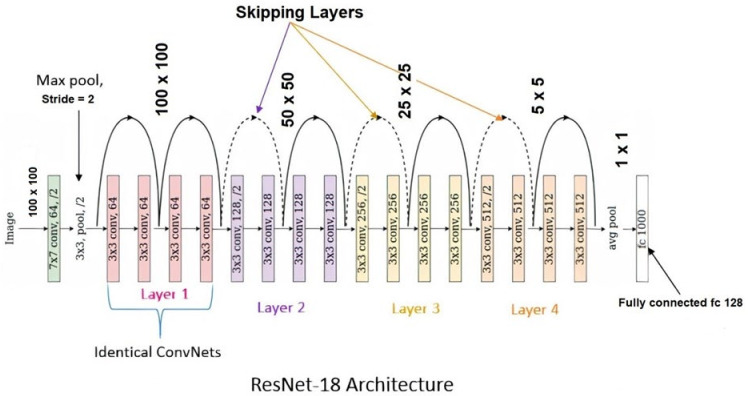
Detail Layer Architecture Representation of ResNet18 Deep Learning Neural Network Model [30].

In order to obtain segmented lung regions of the CXR images, we used pulmonary contour masks [27,28], and FC-DenseNet103 semantic segmentation algorithm [29] as depicted in Figure 2. Initially, the figures consisted of many unwanted regions outside the lung contour. As the convolution neural network model does the feature engineering based on the input images fed to the system, it is always important to use the most designed region. It could be done only by the use of the segmentation network. In our model, segmentation helped to get the lung contours which is our region of interest. It not only allows the Deep Learning Model to accurately learn the actual infected area but also makes the model more robust. Thus, our model was fed with the segmented lung contours only.

### 2.3. Processes Used in the Deep Learning Model (DLM)

Figure 1 is a representation of the steps involved in the current DLM classification model. For this process, we used different kinds of deep neural network architecture to improve the model which can filter out the error and generate the optimum results. As the first step, the original images are fed to the system, then after preprocessing, we fed those images for segmentation. After segmentation, we fed the segmented lung contour to the deep neural network model and finally got the classification result.

### 2.4. Description of Deep Learning Models

Because of segmentation, the locality of the interest area is more exposed to the deep neural network model. It helps to get the understanding of the severity of the pulmonary lesions by visualizing the affected areas and damages of lung tissues as appeared from CXRs. Thus, it helps to detect the disease severity and its prognosis.

Our DLM model is an extract of different well known deep learning pretrained models, such as ResNet18 [30], AlexNet [31], DenseNet [32], VGG16 [33] etc. These are well known Deep Learning Models or the benchmark deep learning neural network which provide standard trained values. These pretrained models get trained values using the most reliable ImageNet dataset, which contains a total of 14,197,122 images. The ImageNet Large Scale Visual Recognition Challenge (ILSVRC) is a benchmark which helps in developing new models. 

In the model, it is a deep learning code which was taking the optimum vector components to generate the model. ResNet18 is a pre-trained model which has been used in our proposed DLM to enhance the efficiency. We used the optimum value, especially the learned weightage parameter values of ResNet18. The pneumonia study of Rajpurkar et al. [34], who developed a deep learning, convolutional neural network model using CXR images prior to the COVID-19 pandemic inspired our research. The model [34] had a Receiver Operating Characteristic Curve (ROC) value of 0.76 in a multiclass classification model using 14 types of pneumonia. In our study, we also used the concept of multiclass classification model with 3 classes.

**Figure 3:**
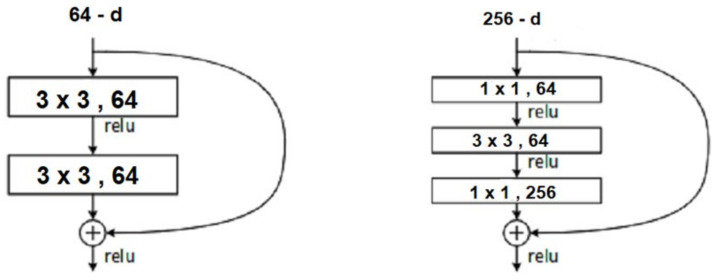
ResNet 2 and ResNet 3-layer block.

ResNet18, used in our model, introduces the concept of skip connection in the deep layers which helps to solve the problem of vanishing gradient. Each ResNet block is either 2-layers deep used in small networks (such as ResNet 18, 34) or 3-layers deep (such as ResNet 50, 101, 152), as shown in Figure 3.

### 2.5. Training, Validation, Testing, and Augmentation of Images

**Figure 4:**
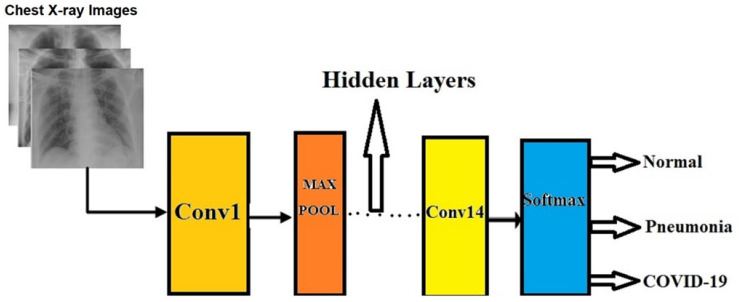
Proposed Deep Learning Model (DLM) Architecture.

The inputs used to train and test the system were those lung X-rays and the outputs were classified three groups: COVID-19, pneumonia (but not COVID positive), or normal cases (not COVID-19 or pneumonia) (Figure 4). The original CXR images were resized to 224 × 224 to be compatible with the pretrained model used in this research. All the images in the dataset used for training, validation, and testing were leveled as 0 (COVID-19 positive), 1 (Pneumonia, but not COVID-19 positive) and 2 (Normal). We had used data augmentation to increase each data sample. It is a machine learning teaching which uses vertical and horizontal flip and increase the data sample with the deep learning framework.

The images were horizontally flipped, magnified, and rotated for augmentation. After all these processes, we had an increased amount of data image samples in training, validation, and testing, while maintaining the balancing property of the sample.

The DLM model used in our work uses ResNet18 Deep Learning Model’s pretrained weightages. It helps a lot for training processes, basically minimizing the forward passes in the deep learning network. Our Deep Learning Model as shown in Figure 4 consists of 46 layers. 14 convolution layers, 23 hidden layers, 4 Max-pooling layers, 2 average polling layers, 2 dropout layers, and one SoftMax layer. The main deep layers are divided into two groups: one group of layers perform operation related to ‘feature learning’ and another group of layers are used for classification. In feature learning, there are a lot of concepts which are used depending upon the research. Our proposed DLM uses deep convolution layers, which consist of a set of convolution operation and pooling operation. In the classification part of the model, there are flatten layers, fully connected layers, and the activation function to get the desired output.

### 2.6. Definitions and Statistical Formulae

The term “sensitivity” (Table 1) tells us out of the total number of people who have the disease (e.g., COVID-19), the number that are correctly classified as having the disease by the model. Specificity of the model is its ability to determine the healthy cases correctly. Positive predictive value (or precision) of the model means out of the total who are detected as disease positive (such as COVID-19 positive) by the model, how many of them are in fact disease positive. Accuracy of the model is the total persons correctly identified (true diseases positive plus true disease negative) out of the total people tested. F1 score is a harmonic mean of two factors i.e., precision and recall (or sensitivity). The performance of the model was measured by several factors including sensitivity, specificity, accuracy and F1 score. False positive fraction and true positive fraction were used to demonstrate the ROC curve.

**Table 1:** Calculation of the Screening Test Statistics.

	Based on the Gold Standard		
	Disease Present	Disease Absent	Total
Predicted Model Positive	True positive (TP)	False positive (FP)	TP + FP
Predicted Model Negative	False negative (FN)	True negative (TN)	FN + TN
Total	TP + FN	FP + TN	TP + FP + FN + TN

$$\mathrm{Sensitivity}\;\mathrm{or}\;\mathrm{Recall}=\frac{\mathrm{TP}}{\left(\mathrm{TP}+\mathrm{FN}\right)}$$

$$\mathrm{Specificity}=\frac{\mathrm{TN}}{\left(\mathrm{FP}+\mathrm{TN}\right)}$$

$$\mathrm{Positive}\;\mathrm{Predictive}\;\mathrm{Value}\;\mathrm{or}\;\mathrm{Precision}=\frac{\mathrm{TP}}{\left(\mathrm{TP}+\mathrm{FP}\right)}$$

$$\mathrm{Accuracy}=\frac{\left(\mathrm{TP}+\mathrm{TN}\right)}{\left(\mathrm{TP}+\mathrm{FP}+\mathrm{FN}+\mathrm{TN}\right)}$$

$$\mathrm{The}\;\mathrm{weighted}\;\mathrm{average}\;\mathrm{of}\;\mathrm{precision}\;\mathrm{and}\;\mathrm{recall},\;\mathrm{known}\;\mathrm{as}\;F1\;\mathrm{Score}=\frac{2\left(\mathrm{Recall}*\mathrm{Precision}\right)}{\left(\mathrm{Recall}+\mathrm{Precision}\right)}$$

$$\mathrm{False}\;\mathrm{Positive}\;\mathrm{Fraction}=\frac{\mathrm{FP}}{\left(\mathrm{FP}+\mathrm{TN}\right)}$$

$$\mathrm{True}\;\mathrm{Positive}\;\mathrm{Fraction}=\frac{\mathrm{TP}}{\left(\mathrm{TP}+\mathrm{FN}\right)}$$

### 2.7. ROC Curve

A Receiver Operating characteristic Curve or ROC curve is a graphical representation of the performance of a classification model. This curve used True Positive Rate (TPR) (sensitivity) in the y-axis, and False Positive Rate (FPR) (1—specificity) in the x-axis. The value of the area under the curve (AUC) ranges from 0 to 1, of which a value of 0 means the predictions are 100% wrong, and a value of 1 means predictions are 100% correct. Any value closer to 1.0 is a very accurate model. 

### 2.8. Confusion Matrix

A Confusion matrix is a table showing the number of cases in each classification that were predicted correctly by the model out of the total cases in the target class. In our study, the represents a 3 × 3 matrix because we used three classifications in the model: COVID-19, pneumonia, and healthy cases.

## 3. Results

Our proposed model used a large dataset of 10,040 samples, of which 2143 had COVID-19, 3674 had pneumonia, and 4223 were normal (not COVID-19 or pneumonia). Due to augmentation, there are horizontal, vertical, and rotational flip; in these processes the image sample was increased by 65% as shown in Table 2. 

Of the dataset, 13,251 (80%) were used for training the model and the remaining 3313 (20%) were used for testing the model. From the training dataset, 1325 images (10%) were used for validation.

### 3.1. Validity Data of the DLM Model

The proposed Deep Learning (DLM) model had a detection accuracy of 96.43% and a sensitivity of 93.68%. We have trained all models using batches of size 50 for 200 epochs using the Stochastic Gradient Descent (SGD) method to reduce the loss function. 

**Figure 5:**
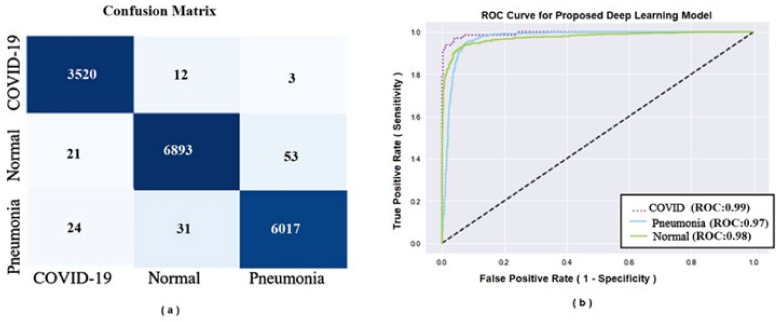
(**a**) Confusion matrix of the sample data; (**b**) Area under the ROC curve for the model.

Figure 5 shows the confusion matrix (left) and AUC (right). The AUC was 99% for COVID-19, 97% for pneumonia, and 98% for normal cases. 

From the Figure 5a, the confusion matrix shows that out of 3535 COVID-19 cases 3520 were correctly detected and 12 images detected as a normal cases and 3 as pneumonia cases. Out of 6967 normal cases, 6893 were detected correctly, 21 detected as a COVID-19 and 53 detected as pneumonia. Also, out of 6062 pneumonia cases, 6017 were detected correctly but 24 was detected as COVID-19 and 31 as normal cases. From the Figure 5b, the AUC clearly shows the high prediction rates for the DLM model.

### 3.2. Comparison of Performance

A comparison of performance, in terms of accuracy, sensitivity, specificity, and F1-score of our proposed DLM model and other models is presented in Table 3. We analyzed some of the previous research data and showed a comparative analysis with the proposed model in the table. The research data reported here were published since mid-2020. The number of CXR images used was not available in one study [35]. Two studies [36,37], which consisted of large CXR samples, used a deep convolutional neural network model, whereas another study with over 2900 samples [38] used a hybrid model. Compared to the other models, the proposed DLM model showed some improvement in all parameters of performance matrices, including accuracy, sensitivity, specificity and F1-score (weighted average of precision and recall).

## 4. Discussion

The proposed DLM model in this study was efficient in the screening of cases with COVID-19, pneumonia, and normal (not COVID-19 or pneumonia). The area under the ROC curves in cases who had COVID-19 (0.99), pneumonia (0.97), or were normal (0.98) showed high accuracy of the results as predicted by the DLM model.

As indicated by the performance indicators, such as accuracy, sensitivity, specificity and F1-score, the proposed DLM outperformed all other models. The ROC curve also showed the high prediction rates for COVID-19, pneumonia, and health states in our DLM model. A similar high accuracy of 99% was reported by a recent study [43], which was achieved by enhanced VGG16 in the detection of CXR images from COVID-19 and pneumonia patients. Several reports have emphasized the importance of using CXR images in the COVID-19 diagnosis using AI [44,45,46,47]. 

The Convolutional Neural Network (CNN) is the one of the most prominent domain of AI and machine learning (ML). It is widely used in different types of classification problem as well as in image classification problems [48,49,50]. Rahaman and colleagues (2020) used 15 different CNN models in a transfer learning process [51]. Another study using the DenseNet201 model [52] yielded an overall accuracy of 92% with a sensitivity of 94% for COVID-19 screening using chest X-rays. 

Due to the advancement of different deep neural network, such as ResNet18, ResNet50 [30], AlexNet [31], DenseNet [32], and VGG16 [33], we extracted techniques from these models, and were able to make our model more error-free, sophisticated, and accurate. Similar to our study, another recent study [53] applied the method of preprocessing for lung segmentation, which removed the unwanted surroundings of the lung X-rays and kept the desired lung tissues only. By this process, the authors achieved a high detection accuracy rate of 97% for COVID-19. We used the similar process of preprocessing and then trained the classification model for the learning scheme. We thoroughly evaluated the problems associated with data imbalance in some of the previously reported studies [54,55,56,57,58]. Wang et al. [36] developed a machine learning model, called COVID-Net using 13,975 CXR images. They had a total of 8066 normal cases and 5538 patients with non-COVID-19 pneumonia, whereas, they had only 266 COVID-19 patient cases. This makes their dataset having the issue of data imbalance. Minaee et al. [40] developed their model using 5000 CXR images. They used a transfer learning technique to use the pretrained weightages in their model. The main disadvantages of this kind of transfer learning weightages is that the original deep learning network did not actually use medical dataset, more specially COVID-19 dataset; so the weightages may lead to bias prediction to some extent.

Prior to COVID-19, Rajpurkar et al. [34] described a model named as CheXNet, where they used deep neural network to classify 14 kinds of pneumonia disease and a large number of samples (more than 100,000 X-rays) for the model. It was a very promising research work which could greatly influence the study of COVID-19 research using ML. However, several other studies suffered from issues of low samples. For example, Khan et al. [41] used CoroNet, which is a deep convolutional neural network model. The model consisted of 310 normal images, 284 images with COVID-19, 327 cases with viral pneumonia, and 330 cases with bacterial pneumonia. The problem of small sample size was identified in some other studies [42]. If we begin with very few COVID-19 samples and comparatively large number of CXR images for normal and pneumonia samples, the data are not balanced, and the weights of the other samples may lead to biased predictions. Our study included a large sample of 10,040 which increased to 16,574 after augmentation. 

### 4.1. Limitations

Most of the model built are limited with the amount of COVID-19 images, especially at the beginning of the pandemic because of unavailability or limited access to publicly available records. For this reasons, we initially could retrieve only 2143 number of COVID-19 images which was further increased to 3535. A limitation of the study is the lack of validation, i.e., use of the program in a different setting or context.

### 4.2. Strengths

In this study, chest X-rays were preferred over chests CT scan images. The reason behind this was that X-ray machines are readily available in most of the hospitals. Also, the CT scan machines are costlier that X-ray machines. The number of CXR images in our study was quite large to accurately measure the model performances. The performance indicators such as accuracy, sensitivity, specificity, and F1 scores of the study model were high. 

## 5. Conclusions

In this research work, an automated analysis of CXR was achieved using deep learning-based approaches. The model had a high sensitivity, specificity, and accuracy regarding the identification of cases with COVID-19, pneumonia, and normal subjects. The results of high performance of the model make it a useful tool to screen COVID-19 cases using CXR. Promising results of the DLM model indicate that this model can be helpful for the medical professionals globally. The research potential of this study is of significant interest as we are still living in the pandemic scenario and new variants of COVID-19 are emerging as substantial threats of infectiousness and/or virulence. Should we have automated detection techniques available and health professionals get the information in a timely manner, the diagnosis and the management of COVID-19 will be more efficient. 

## Figures and Tables

**Table 2:** Dataset Sample details before and after data augmentation.

Stage of Data	COVID-19	Pneumonia	Normal	Total
Before augmentation	2143	3674	4223	10,040
After augmentation	3535	6072	6967	16,574

**Table 3:** Comparison of accuracy, sensitivity, specificity, and F1 score of the proposed model and other models.

Study [Ref]	No. Images Used	AI Method Used	Accuracy	Sensitivity	Specificity	F1-Score
Proposed Model	10,040	Deep Learning Model	96.43	93.68	99.0	93.0
Das et al. [35]	Unknown	Deep transfer learning	92.41	91.29	92.0	89.0
Wang et al. [36]	13,975	Deep convolutional neural network	92.04	90.41	94.0	87.0
Narin et al. [37]	7486	Deep convolutional neural network	91.26	89.24	93.0	86.0
Altan et al. [38]	2905	A hybrid model, having 2D Curvelet transformation, a Salp swarm algorithm (SSA) and deep learning	91.85	92.42	91.0	90.0
Ozturk et al. [39]	1000	Deep neural network	93.40	92.12	89.0	90.0
Minaee et al. [40]	5,000	Deep transfer learning	90.49	92.08	91.0	88.0
Khan et al. [41]	1300	Deep neural network	89.60	84.98	87.0	86.0
Civit-Masot et al. [42]	396	VGG16-based (convolutional) Deep Learning Model	94.52	92.11	96.0	92.0

## Data Availability

The model, data, and methods used in the research are presented in sufficient detail so that other researchers can replicate the work. The secondary data for this study are extracted from publicly available sources.
